# Current strategies to induce selective killing of HIV‐1‐infected cells

**DOI:** 10.1002/JLB.4MR0422-636R

**Published:** 2022-06-16

**Authors:** Grant R. Campbell, Stephen A. Spector

**Affiliations:** ^1^ Department of Pediatrics Division of Infectious Diseases University of California San Diego La Jolla California USA; ^2^ Division of Infectious Diseases Rady Children's Hospital San Diego California USA

**Keywords:** HIV‐1, IAP, cell death, SMAC mimetics, autophagy, apoptosis, autosis

## Abstract

Although combination antiretroviral therapy (ART) has led to significant HIV‐1 suppression and improvement in immune function, persistent viral reservoirs remain that are refractory to intensified ART. ART poses many challenges such as adherence to drug regimens, the emergence of resistant virus, and cumulative toxicity resulting from long‐term therapy. Moreover, latent HIV‐1 reservoir cells can be stochastically activated to produce viral particles despite effective ART and contribute to the rapid viral rebound that typically occurs within 2 weeks of ART interruption; thus, lifelong ART is required for continued viral suppression. Several strategies have been proposed to address the HIV‐1 reservoir such as reactivation of HIV‐1 transcription using latency reactivating agents with a combination of ART, host immune clearance and HIV‐1‐cytotoxicity to purge the infected cells—a “shock and kill” strategy. However, these approaches do not take into account the multiple transcriptional and translational blocks that contribute to HIV‐1 latency or the complex heterogeneity of the HIV‐1 reservoir, and clinical trials have thus far failed to produce the desired results. Here, we describe alternative strategies being pursued that are designed to kill selectively HIV‐1‐infected cells while sparing uninfected cells in the absence of enhanced humoral or adaptive immune responses.

## Graphical Abstract

Overview of current strategies to kill HIV‐1‐infected cells.

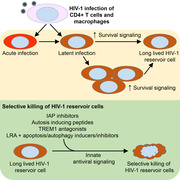

**Abbreviations**
AKT1: AKT serine/threonine kinase 1
AIF: apoptosis‐inducing factor
ART: antiretroviral therapy
ATG: autophagy related
BAD: BCL2‐associated agonist of cell death
BAK1: BCL2 antagonist/killer 1
BAX: BCL2‐associated X, apoptosis regulator
BCL2: BCL2 apoptosis regulator
BCL2A1: BCL2‐related protein A1
BCL2L11: BCL2 like 11
BCL2L12: BCL2 like 12
BCL2L13: BCL2 like 13
BCLXL: B‐cell lymphoma‐extra large
BECN1: beclin 1
BID: BH3 interacting domain death agonist
BIRC: baculoviral IAP repeat containing
BOK: BCL2 family apoptosis regulator BOK
DDX3: DEAD‐box helicase 3
DIABLO: diablo IAP‐binding mitochondrial protein
DISC: death‐inducing signaling complex
ER: endoplasmic reticulum
FADD: Fas‐associated via death domain
FAS: Fas cell surface death receptor
GLIPR2: GLI pathogenesis related 2
HRK: harakiri BCL2 interacting protein
HDAC: histone deacetylase
HDACi: histone deacetylase inhibitor
IAP: inhibitor of apoptosis protein
LRA: latency reversing agent
LUBAC: linear ubiquitin chain assembly complex
MATR3: matrin 3
MDM2: MDM2 proto‐oncogene
MOMP: mitochondrial outer membrane permeabilization
MTOR: mechanistic target of rapamycin kinase
PLGA: poly(lactic‐*co*‐glycolic acid)
PLWH: persons living with HIV‐1
RIPK: receptor interacting serine/threonine kinase
SMAC: second mitochondria‐derived activator of caspase
SQSTM1: sequestosome 1
TRADD: TNFRSF1A‐associated via death domain
TNFRSF: TNFR superfamily
TNP: CD4+ T cell plasma membrane wrapped poly(lactic‐*co*‐glycolic acid) core nanoparticles
TREM1: triggering receptor expressed on myeloid cells 1
ULK: unc‐51 like autophagy activating kinase
v‐FLIP: viral FADD‐like IL‐1β‐converting enzyme‐inhibitory protein
WIPI: WD‐repeat protein interacting with phosphoinositides
XIAP: X‐linked inhibitor of apoptosis
ZFYVE1: zinc finger FYVE‐type containing 1

## 1 INTRODUCTION

HIV‐1 infects and establishes a productive infection in CD4+ T cells, macrophages, microglia, and hematopoietic stem cells during the acute phase of infection through the integration of replication‐competent proviruses into, for the most part, actively expressed genes in the host genome.^1^, ^2^, ^3^, ^4^ Despite much research, the precise molecular mechanisms that govern whether an infected cell is destined to become latently (harbors replication‐competent virus but does not produce infectious viruses unless activated) or productively infected are unknown. Moreover, the mechanisms of latency may vary both between persons living with HIV‐1 (PLWH) and between cells within a single patient. A number of epigenetic modulators have been proposed to be responsible for latency, most of which work to maintain a transcriptionally silent chromatin architecture at the HIV‐1 promoter,^5^, ^6^ but other studies do not support these findings.^7^ These studies suggest that viral transcription still occurs during latency, and that the principal blocks to HIV‐1 transcription in vivo are downstream and inhibit proximal elongation, distal transcription, polyadenylation, splicing, and the nuclear export of viral RNAs, which require host cellular machinery working in concert with HIV‐1 proteins to overcome.^5^, ^7^, ^8^ As an example, circulating resting CD4+ T cells, the largest and best characterized HIV‐1 reservoir, have been found to express low levels of matrin 3 (MATR3), an essential cofactor for HIV‐1 Rev‐mediated RNA export,^9^ and express high levels of cellular microRNAs (miR‐28, miR‐125b, miR‐150, miR‐223, and miR‐382) that bind to the 3′ untranslated region of viral mRNAs, inhibiting their translation.^10^

It is generally accepted that during untreated progressive infection, HIV‐1 continually replicates in secondary lymphoid tissues.^11^ In contrast, there is much debate regarding ongoing viral replication under suppressive antiretroviral therapy (ART).^11^, ^12^, ^13^, ^14^, ^15^, ^16^, ^17^, ^18^, ^19^ However, cell‐associated RNA transcripts are detectable in PLWH receiving fully suppressive ART that have undetectable replication competent viremia,^7^, ^16^, ^19^ and the virus remains abundant in lymphatic tissues where ART concentrations may not reach therapeutic levels, and where HIV‐1 appears to infect new cells.^18^

Although HIV‐1 establishes a productive infection in CD4+ T cells, macrophages, microglia, and hematopoietic stem cells, there is also much debate as to which cells constitute the HIV‐1 reservoir—the cell population that allows replication‐competent HIV‐1 to persist for years despite suppressive ART. Although latent HIV‐1‐infected resting CD4+ T cells were thought to be the only population to fulfill this role,^20^ accumulating evidence suggests that hematopoietic stem and progenitor cells,^21^ macrophages, and microglia^22^, ^23^ are all also distinct HIV‐1 reservoirs. A number of factors are thought to account for the stability and persistence of the HIV‐1 reservoir despite effective antiviral immunity and/or ART.^24^ These include the viral cytopathic effect, immune clearance, and clonal expansion of latent HIV‐1‐infected CD4+ T cells.^25^, ^26^ The precise mechanism(s) by which latent HIV‐1‐infected cell clonal expansion occurs are not completely understood, but are thought to include general immune activation, antigen driven expansion and contraction of infected CD4+ T cell clones, homeostatic proliferation, and HIV‐1 integration site‐dependent provirus‐driven clonal expansion.^4^, ^11^, ^19^, ^27^, ^28^, ^29^, ^30^, ^31^, ^32^, ^33^

Although ART suppresses HIV‐1 replication, improves immune function, reduces comorbidities, and has led to significant improvements in both longevity and quality of life in PLWH, it does not completely reverse the significant loss in virus‐specific immune functions observed in PLWH,^34^ nor does it eliminate the preexisting HIV‐1 reservoir.^24^ Additionally, ART presents challenges such as adherence to drug regimens, the emergence of resistant viruses, and cumulative ART toxicity. Moreover, the latent cells that remain are a source of viral resurgence upon ART interruption or failure.^17^, ^35^, ^36^ Thus, a strategy to cure HIV‐1 is an urgent and unmet need. Although cure has been achieved in a few persons, in each of these cases patients underwent allogeneic hematopoietic stem‐cell transplantation with HIV‐1 resistant CCR5Δ32/Δ32 CD34+ peripheral blood stem cells.^37^, ^38^ Although this procedure is logistically not unfeasible for deployment across the almost 38 million PLWH who do not have other conditions that make them candidates for such a procedure, it demonstrates that a cure is possible.

## 2 SHOCK AND KILL

**FIGURE 1:**
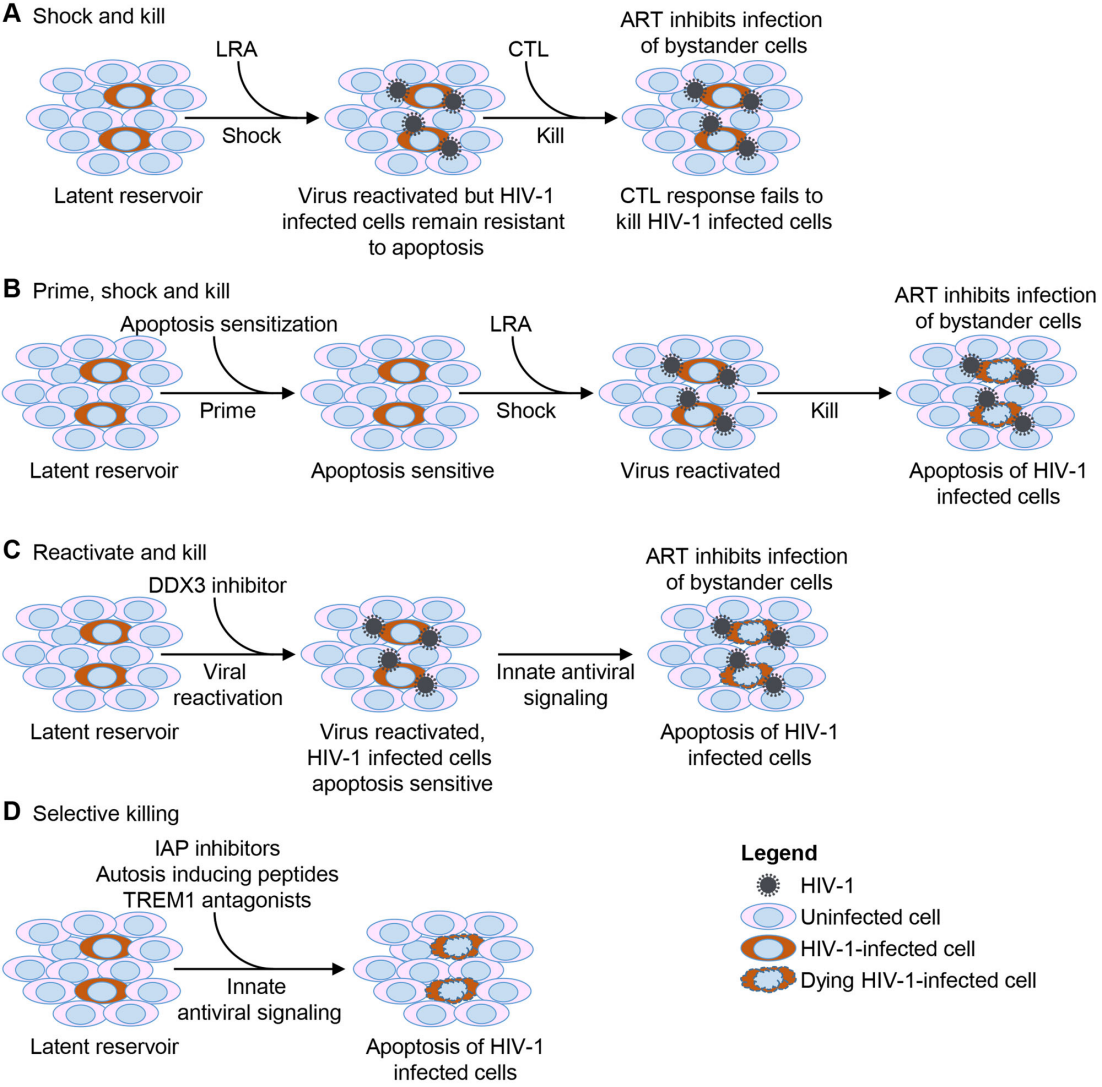
Selective killing of HIV‐1‐infected cells. (A) The “shock and kill” strategy aims to decrease the HIV‐1 reservoir through the reactivation of proviral transcription using latency reversing agents (LRA), leading to the elimination of infected cells by CTLs and HIV‐1 cytopathogenesis. (B) The “prime, shock, and kill” is similar to the “shock and kill” strategy with the addition of pharmacologic agents to overcome the latent reservoir cells inherent resistance to cell death. (C) This strategy simultaneously reactivates the expression of viral RNA while also increasing the infected cells sensitization to innate antiviral signaling pathways. (D) IAP inhibitors, autosis inducing peptides, and TREM1 antagonists can all induce death in HIV‐1‐infected cells without the need for reactivation of the virus with minimal toxicity to uninfected cells

The most prominent approach to achieve a HIV‐1 cure is the “shock and kill” strategy. This strategy attempts to induce the reactivation of HIV‐1 transcription from latent HIV‐1‐infected CD4+ T cells using latency reversing agents (LRAs) such as histone posttranslational modification modulators, TLR agonists, NF‐κB stimulators, nonhistone chromatin modulators, extracellular stimulators, and mechanistic target of rapamycin kinase (MTOR) activators^39^ (the latter being essential to reactivate HIV‐1 from latency^40^) with the expectation that the activation of the integrated virus will result in an increase in the expression of viral proteins and virions such that they will be killed through either immune mediated clearance or HIV‐1‐mediated cytolysis (Figure 1(A)). This approach does not aim to clear the majority of infected cells—98% of HIV‐1 proviruses in resting CD4+ T cells are defective^41^—only resting CD4+ T cells that contribute to the HIV‐1 reservoir. Unfortunately, the current evidence from clinical trials have shown that although some LRA are able to induce viral transcription in some cell types, this alone is insufficient to reduce the size of the HIV‐1 reservoir.^7^, ^39^, ^42^, ^43^, ^44^, ^45^, ^46^ A possible explanation for this comes from ex vivo studies that suggest that no currently tested non‐T cell‐activating LRA can overcome each of the blocks in proximal elongation, distal transcription, polyadenylation, splicing, and the nuclear export of viral RNAs,^7^, ^42^ nor do they increase the expression of MATR3.^9^ As an example, although the histone deacetylase (HDAC) inhibitors (HDACi) panobinostat and romidepsin both increase total and elongated HIV‐1 transcripts, they have little effect on polyadenylated or multiply spliced transcripts.^7^ Given the fundamental importance of polyadenylation to HIV‐1 RNA nuclear export, stability, and translation into HIV‐1 proteins,^47^, ^48^ which are essential for both productive infection and T cell recognition and killing, it is likely that although these agents will increase cell‐associated HIV‐1 RNA, this will be insufficient to induce productive infection, immune‐mediated clearance, and/or HIV‐1‐mediated cytolysis. Moreover, both panobinostat and romidepsin, as well as the most studied LRA to date, vorinostat, can suppress the CTL response and impair their ability to kill HIV‐1‐infected CD4+ T cells.^49^ In vitro studies also suggest that HDACi suppress the IFNγ response to HIV‐1 antigens, impair natural killer cell function, increase the permissiveness of uninfected primary CD4+ T cells to HIV‐1 infection, and are potentially cytotoxic over long durations.^50^, ^51^, ^52^ However, to date, these concerns have not been observed during short courses of treatment in vivo.^53^, ^54^, ^55^ Similarly, there is no evidence that LRA can alone overcome the inherent intrinsic resistance of latent HIV‐1‐infected cells to apoptotic stimuli, and in the case of protein kinase C activators, can actually increase the expression and/or activation status of antiapoptotic regulatory proteins.^56^, ^57^ Additionally, LRA may have different effects in HIV‐1‐infected macrophages. For example, belinostat, givinostat, vorinostat, panobinostat, romidepsin (all HDACi), and JQ1 (a BET bromodomain inhibitor) all inhibit MTOR complex 1 in vitro in HIV‐1‐infected macrophages, inducing autophagy and an autophagy‐dependent decrease in both HIV‐1 release from infected macrophages and cell‐associated HIV‐1 capsid abundance in the absence of cell death.^58^, ^59^ Current research is now examining the “shock and kill” approach in the context of supporting strategies that include broadly neutralizing or engineered bispecific antibodies, therapeutic vaccines, chimeric antigen receptors, or checkpoint inhibitors. However, the results from recent trials using this strategy have not been promising.^53^, ^55^, ^60^ Fundamentally, these supporting strategies still do not address each of the multiple blocks in viral transcription and translation such that they do not induce outgrowth of latent HIV‐1 to elicit immune mediated clearance. Moreover, they do not address the heterogeneous HIV‐1 reservoir in various tissue compartments and sanctuary sites, the homeostatic proliferation of latently infected cells, or the presence of noninducible HIV‐1.^61^ Thus, eradication of the HIV‐1 reservoir using a “shock and kill” strategy might be very difficult to achieve.^55^

## 3 SELECTIVE KILLING OF HIV‐1‐INFECTED CELLS

Acute HIV‐1‐infected CD4+ T cells are susceptible to dying through direct viral cytopathogenesis, superantigen‐induced cell death, CTL‐mediated killing, antibody‐dependent cell cytotoxicity, and/or syncytia formation. In vitro studies of cell line and primary cell models of latent HIV‐1 infection suggest changes in the transcriptional profile of these cells to produce more endogenous apoptosis regulatory proteins including CSF1 (M‐CSF), CFLAR (c‐FLIP), triggering receptor expressed on myeloid cells 1 (TREM1), BCL2 family members including BCL2 and BCLXL, and the inhibitor of apoptosis proteins (IAP) XIAP, baculoviral IAP repeat containing (BIRC)2 (cIAP‐1), BIRC3 (cIAP‐2), and BIRC5 (survivin), decreases the expression and/or inactivates proapoptotic proteins including BCL2‐associated agonist of cell death (BAD) and BCL2‐associated X, apoptosis regulator (BAX), and activates the PI3K/AKT1 cell survival pathway leading to an apoptosis resistant phenotype.^62^, ^63^, ^64^, ^65^, ^66^, ^67^, ^68^, ^69^, ^70^ Moreover, HIV‐1 has evolved to evade autophagic degradation and suppress the macroautophagy‐dependent immune responses.^71^, ^72^, ^73^, ^74^ Thus, strategies that target the increased expression of antiapoptosis regulatory proteins to trigger pharmacologically and specifically regulated cell death pathways in infected cells while sparing uninfected bystander cells could be of interest (Figures 1(B), 1(C), and 1(D)).

### 3.1 Apoptosis

**FIGURE 2:**
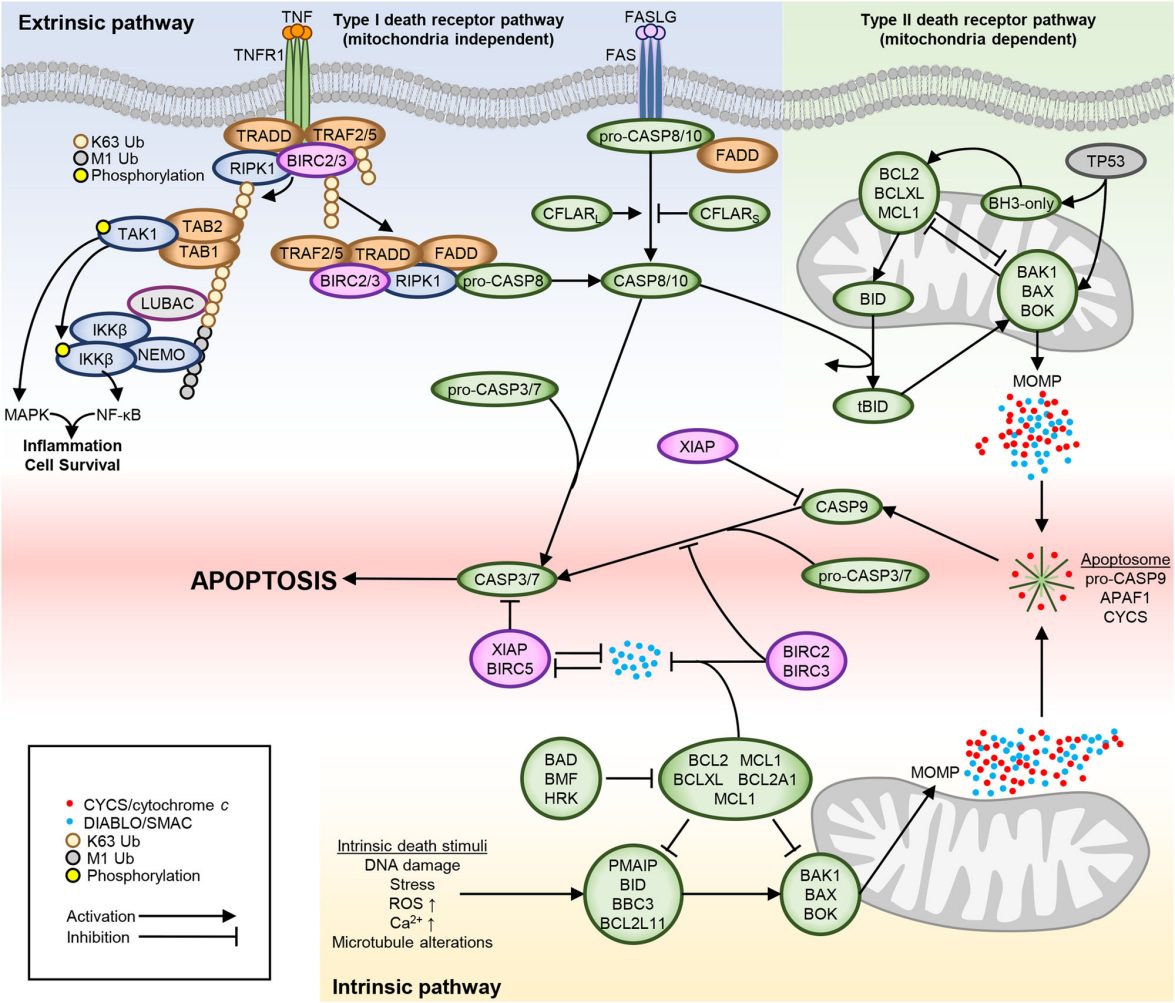
Regulation of apoptosis. The initiation of apoptosis is a multistep process that can be either extrinsic or intrinsic. In the extrinsic pathway, cognate ligand binding to TNFRSF members can either promote the direct recruitment of TRADD (as is the case for TNFRSF25 or TNFR1) or FADD (as is the case for FAS and TNFRSF10A [TRAILR1]). In the case of TRADD recruitment, this either promotes the recruitment of FADD leading to a caspase signaling cascade resulting in the activation of caspase‐3 and apoptosis, or TRADD recruits RIPK1 and TRAF2, triggering NF‐κB activation, cell survival and a proinflammatory response. In both cases, the extrinsic pathway can be either mitochondria independent (type I death receptor pathway) or converge with the intrinsic pathway and be mitochondria dependent (type II death receptor pathway) when BID is activated by caspase‐8, which then oligomerizes BAK1 leading to mitochondrial outer membrane permeabilization (MOMP) and the release of cytochrome c from the mitochondrial intermembrane space. The key event in the intrinsic pathway is the formation of the apoptosome and subsequent activation of caspase‐9 after MOMP. Also released during MOMP are DIABLO/SMAC, which promotes apoptosis indirectly by inhibiting IAPs, and AIF, which promotes parthanatos, a caspase‐independent form of cell death (not shown)

There are 2 general pathways towards apoptosis, the extrinsic pathway and the intrinsic pathway (Figure 2)^75^, ^76^ These pathways are neither mutually exclusive nor independent of one another as they converge in the final phase to induce cell death. The extrinsic pathway is triggered by changes in the extracellular environment that can activate either transmembrane dependence receptors such as DCC netrin 1 receptor and patched 1 or transmembrane TNF receptor superfamily (TNFRSF) death receptors that possess an intracellular death domain, such as TNFR1, and Fas cell surface death receptor (FAS). Dependence receptor‐induced apoptosis has implications in cancer cell survival as they induce caspase lethal signaling cascades when their cognate trophic ligand availability is below a defined level.^77^ The TNFRSF death receptors interact with their specific ligands, leading to the recruitment of the adaptor proteins Fas‐associated via death domain (FADD) and TNFRSF1A‐associated via death domain (TRADD) to their cytoplasmic death domain, resulting in a signaling cascade that leads to the formation of a death‐inducing signaling complex (DISC) that activates the initiator caspases caspase‐8 and caspase‐10. These then activate the downstream effector caspases, such as caspase‐3, caspase‐6, and caspase‐7, to effect cell death. However, death receptor activation does not necessarily result in apoptosis. For example, the posttranslational modification status of receptor interacting serine/threonine kinase (RIPK)1 dictates whether TNFR1 activation results in the assembly of prosurvival versus prodeath signaling complexes (Figure 2). Following TNF stimulation, RIPK1 is recruited via a TRADD‐independent manner, followed polyubiquitination by BIRC2, BIRC3, and linear ubiquitin chain assembly complex (LUBAC),^78^ which can promote prosurvival NF‐κB signaling through the assembly of the TNFR1 signaling complex^79^ while also inhibiting the apoptosis/necroptosis inducing catalytic activity of RIPK1 by inhibiting the assembly of distinct complexes that regulate cell death, including FAS signaling.^79^, ^80^, ^81^

The intrinsic pathway is triggered by a variety of perturbations either from intracellular organelles or from the extracellular microenvironment. These stressors include diverse stimuli including DNA damage, replicative, endoplasmic reticulum (ER) or lysosomal stress, reactive oxygen species and Ca^2+^ overload, and microtubule alterations (Figure 2). The critical step for intrinsic apoptosis is the irreversible and widespread mitochondrial outer membrane permeabilization (MOMP), controlled by proapoptotic and antiapoptotic members of the BCL2 family. In response to apoptotic stimuli, the BH3‐only containing BCL2 members including BAD, BCL2 binding component 3 (BBC3) (PUMA), BCL2L11 (BIM), BH3 interacting domain death agonist (BID), BCL2 interacting killer (NBK), BCL2 modifying factor, HRK (DP5), and PMA‐induced protein 1 (NOXA) activate, directly or indirectly, the effector BCL2 members BCL2 antagonist/killer 1 (BAK1) (BAK), BAX, and/or BOK to form toroidal lipidic pores leading to MOMP that releases molecules from within the mitochondria, including cytochrome *c* and the proapoptogenic diablo IAP‐binding mitochondrial protein (DIABLO) (also known as second mitochondria‐derived activator of caspase [SMAC]), into the cytoplasmic space. The antiapoptotic members of the BCL2 family, BCL2, BCL2A1, BCLXL, BCL2L12 (BCLW), and MCL1 apoptosis regulator, BCL2 family member can antagonize this pore‐forming activity. The cytosolic pool of cytochrome *c* binds to apoptotic protease activating factor 1 and procaspase‐9 forming the apoptosome. Caspase‐9 is then activated and catalyzes the proteolytic activation of the downstream effector capsases caspase‐3 and caspase‐7. DIABLO can bind to the BIR domains of IAPs,^82^ thus preventing the XIAP antagonization of caspase‐3, ‐7, and ‐9^83^, ^84^ while also activating the E3 ubiquitin ligase activity of IAPs including BIRC2 and BIRC3 promoting their autoubiquitination and proteasomal degradation.^84^, ^85^ The latter prevents the BIRC2‐ and BIRC3‐mediated ubiquitination of RIPK1, which results in switching the TNFR superfamily signaling from prosurvival to proapoptotic through the formation of a FADD–caspase‐8‐containing complex that leads to caspase‐8 activation.^86^ Moreover, IAPs inhibit FAS ligand‐induced cell death by limiting RIPK1 recruitment to FAS.^80^ Apoptosis‐inducing factor (AIF) is also released from the mitochondria, which can stimulate parthanatos, a caspase independent form of regulated cell death.

### 3.2 Macroautophagy

**FIGURE 3:**
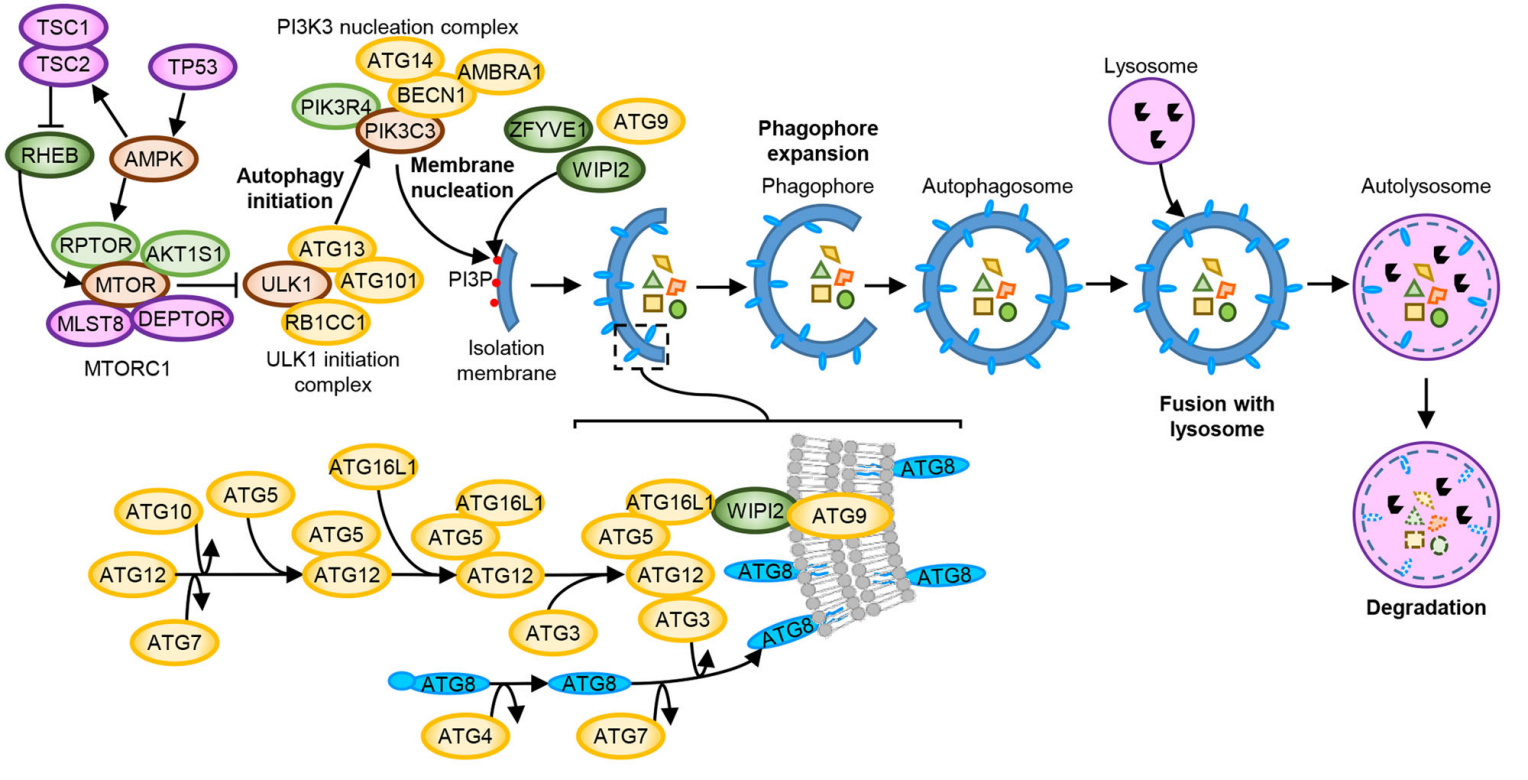
Regulation of autophagy. The initiation of autophagy is a multistep process, the main regulators of which are MTOR, an inhibitor, and AMPK an activator. MTORC1 inhibition drives the formation of the phagophore through the formation of the ULK1 complex that directly activates the PIK3C3 complex. This complex translocates to endoplasmic reticulum sites and produces PI3P, which recruits WIPI2 and ZFYVE1. In the ATG12 conjugation system, ATG12 is covalently attached to ATG5, which is then attached to ATG16L1. WIPI2b acts immediately upstream of ATG16L1 and recruits ATG12–ATG5‐ATG16L1 to PI3P‐tagged phagophores. The ATG12–ATG5‐ATG16L1 complex then promotes conjugation of ATG8 proteins with phosphatidylethanolamine leading to their incorporation into the phagophore membranes where they interact with cargo receptors harboring LC3‐interacting motifs that recruit and incorporate ubiquitin‐decorated cargoes into the nascent autophagosome. After detachment of ATG factors, the phagophore closes through scission forming the autophagosome, which then fuse with lysosomes resulting in the degradation of the engulfed components

Macroautophagy is 1 of 3 principal types of autophagy (the other 2 being microautophagy and chaperone‐mediated autophagy), an evolutionarily conserved major catabolic degradative process that occurs in all eukaryotic cells and is constitutively functional at low levels. Autophagy is up‐regulated under conditions of cellular stress including nutrient deprivation and infection to recycle cytoplasmic components to generate biologic macromolecule monomers and energy, and to remove unneeded and/or damaged organelles and protein complexes to maintain cellular homeostasis and survival, as well as the degradation and elimination of toxic proteins and invasive microorganisms to control infection and inflammation. In addition, autophagy plays a critical role in thymic selection, antigen presentation, cytokine production, lymphocyte homeostasis and survival, and immunometabolism.^87^ In macroautophagy, a dynamic complex signaling pathway initiates the deployment of proteins, lipids, and membranes to form a double‐membrane sequestering phagophore on membrane sites predominately located on ER (other membrane sites have also been implicated).^88^ Unlike the formation of secretory vesicles, phagophore formation, and by extension autophagosome biogenesis, is thought to be a de novo process, although there is still some debate on this point.^89^ Autophagosome biogenesis has 3 main steps: initiation, nucleation, and membrane expansion (Figure 3). The signaling pathway that initiates the formation of autophagosomes requires a number of conserved factors including the unc‐51 like autophagy activating kinase (ULK1) complex (comprising ULK1, ULK2, autophagy related (ATG)13, ATG101, and RB1CC1 [FIP200]), the PIK3C3 (Vps34) complex I (composed of PIK3C3, PIK3R4, Beclin 1 [BECN1], and ATG14), the ATG9/ATG9A cycling system, WD‐repeat protein interacting with phosphoinositides (WIPIs), a conjugation system for Atg12–ATG5 and an ATG8 family lipidation system (Figure 3). In mammalian cells, selectivity is often conferred by cargo receptor proteins such as calcium binding and coiled‐coil domain 2 (NDP52), NBR1 autophagy cargo receptor, optineurin, or SQSTM1 (p62), which act as a link to tether specific cargo to a nascent autophagosome,^90^ or a resident protein expressed on the cargo surface such as BCL2L13 (MIL1) on the surface of mitochondria.^91^ The sequestering phagophore then continues to expand and sequester cargo before closing and forming an autophagosome through membrane scission.^92^ Once formed, the mature autophagosome then fuses with the membrane of lysosomes, forming autolysosomes wherein the autophagic cargo are degraded and/or processed by lysosomal hydrolases (Figure 3).

### 3.3 Autosis

Autosis is a form of autophagy‐dependent cell death that is dependent upon Na+,K+‐ATPase. This is a nonapoptotic, nonnecrotic form of cell death characterized by an increase in autophagosome, autolysosome, and empty vacuole numbers that coincides with the dilation and fragmentation of the ER. This is quickly followed by nuclear membrane convolution, focal ballooning of the perinuclear space, the depletion of ER, autophagosome and autolysosome numbers, swelling of mitochondria, and the focal rupture of the plasma membrane.^93^ The molecular processes that lead to autosis are currently outwith our ken.

### 3.4 Prime, shock, and kill

A number of compounds that target cell death pathways including BCL2 antagonists, PI3K inhibitors, DDX3 inhibitors, polo like kinase 1 inhibitors, and genetic manipulation of long noncoding RNAs with and without an additional LRA have been assessed for their potential to specifically kill reactivated HIV‐1‐infected cells (Figures 1(B) and 1(C)).^56^, ^94^, ^95^, ^96^, ^97^, ^98^ Notably, these approaches all induce selective cell death of HIV‐1‐infected CD4+ T cells with minimal death in uninfected bystander cells. As an example, navitoclax, an inhibitor of BCL2 and BCLXL, when combined with SAR405, an ATP‐competitive inhibitor of PIK3C3, which inhibits autophagy, promotes the selective killing of HIV‐1‐infected CD4+ T cells following latency reversal with ingenol 3,20‐dibenzoate, a protein kinase C activator, in a humanized mouse model of HIV‐1 infection and ex vivo in PBMC from PLWH receiving fully suppressive ART.^56^ This suggests that the inhibition of autophagy combined with BCL2 inhibition may promote cell death selectively in cells with reactivated HIV‐1 expression.^56^ Notably, ingenol 3,20‐dibenzoate is a known T cell activator and proinflammatory cytokine inducer. To counter these adverse effects, the authors added the nonsteroidal anti‐inflammatory veterinary drug carprofen to their drug regimen and this was sufficient to suppress any potential adverse inflammatory effects.

As the immune and inflammatory responses mediated by HIV‐1‐infected microglia and perivascular macrophages contribute to neuronal dysfunction and drive the pathogenesis of HIV‐1‐associated neurocognitive disorders,^99^, ^100^ a strategy that reactivates HIV‐1 and/or leads to an increase in inflammatory cytokines and neurotoxin release is undesirable within this context. Thus, a strategy to eradicate these HIV‐1‐infected cells in the absence of increased viral replication and cellular activation is also of interest, and to purge these reservoirs we must consider alternative approaches (Figure 1(D)). As HIV‐1 infection of macrophages induces the activation of the stress‐related PI3K/AKT1 cell survival pathway, an initial attempt targeted the inhibition of AKT1 in the presence of sodium nitroprusside to promote cell death in both HIV‐1‐infected macrophages and an HIV‐1‐expressing microglia cell line while also restricting viral production.^101^ However, since sodium nitroprusside is a known stimulator of TNF and NO production and has excitotoxicity within the brain parenchyma, this approach could do more harm than good within the central nervous system.

### 3.5 Targeting TREM1

TREM1 is a 30 kDa IgV glycoprotein expressed on the surface of macrophages, microglia, and neutrophils and is up‐regulated during HIV‐1 infection.^66^, ^69^ TREM1 acts synergistically with TLRs and nucleotide oligomerisation domain (NOD)‐like receptors to amplify proinflammatory responses during bacterial and viral infections and to promote cell survival through the induction of BCL2 and mitofusin expression.^102^, ^103^, ^104^ In in vitro HIV‐1‐infected macrophages *TREM1* silencing leads to decreased expression of BCL2, BCLXL, and mitofusin expression and increases the expression of BAD and BAX leading to a significant increase in apoptosis mediated by MOMP, and subsequent cytochrome *c* release and caspase‐9 cleavage with minimal cytotoxicity in uninfected cells.^69^, ^103^

### 3.6 IAP inhibition

Although TREM1 is highly expressed in HIV‐1‐infected macrophages, it is poorly expressed in latent HIV‐1‐infected CD4+ T cells. Conversely, both in vitro latent HIV‐1‐infected CD4+ T cells and macrophages have increased expression of IAPs, which inhibit HIV‐1 transcription,^105^, ^106^ reduce autophagy through the ubiquitination of BECN1^78^ and MDM2 proto‐oncogene (MDM2),^107^ inhibit apoptosis through the antagonization, ubiquitination, and neddylation of apoptosis caspases,^108^, ^109^ and increase the ubiquitination of RIPK1.^78^ DIABLO/SMAC mimetics were developed to inhibit IAPs that are up‐regulated in many forms of human cancers, the up‐regulation of which is associated with chemoresistance, disease progression, and poor prognosis.^110^, ^111^ These synthetic compounds mimic the BIR‐binding N‐terminal tetrapeptide (NH_2_‐AVPI) of DIABLO, and thus bind to and target IAPs for proteasomal degradation. To date, 8 DIABLO/SMAC mimetics (birinapant, LCL‐161, xevinapant, GDC‐0152, CUDC‐427, APG‐1387, HGS1029, and BI 891065) have been tested in patients to determine safety, tolerability, pharmacokinetics, and anticancer activity.^112^ Results so far indicate that they are generally tolerated with limited off‐target effects, but with some dose‐limiting toxicities, and have low efficacy. Future directions in the cancer space include combination therapies with radiation, existing chemotherapies, BCL2 inhibitors, and immunotherapy to improve efficacy. Notably, the DIABLO/SMAC mimetic birinapant selectively kills hepatitis B virus expressing hepatocytes,^113^, ^114^ and treatment of CD4+ T cells or macrophages with DIABLO/SMAC mimetics such as birinapant, LCL‐161, and xevinapant induces the degradation of IAPs leading to the induction of autophagy.^68^, ^70^, ^115^

**FIGURE 4:**
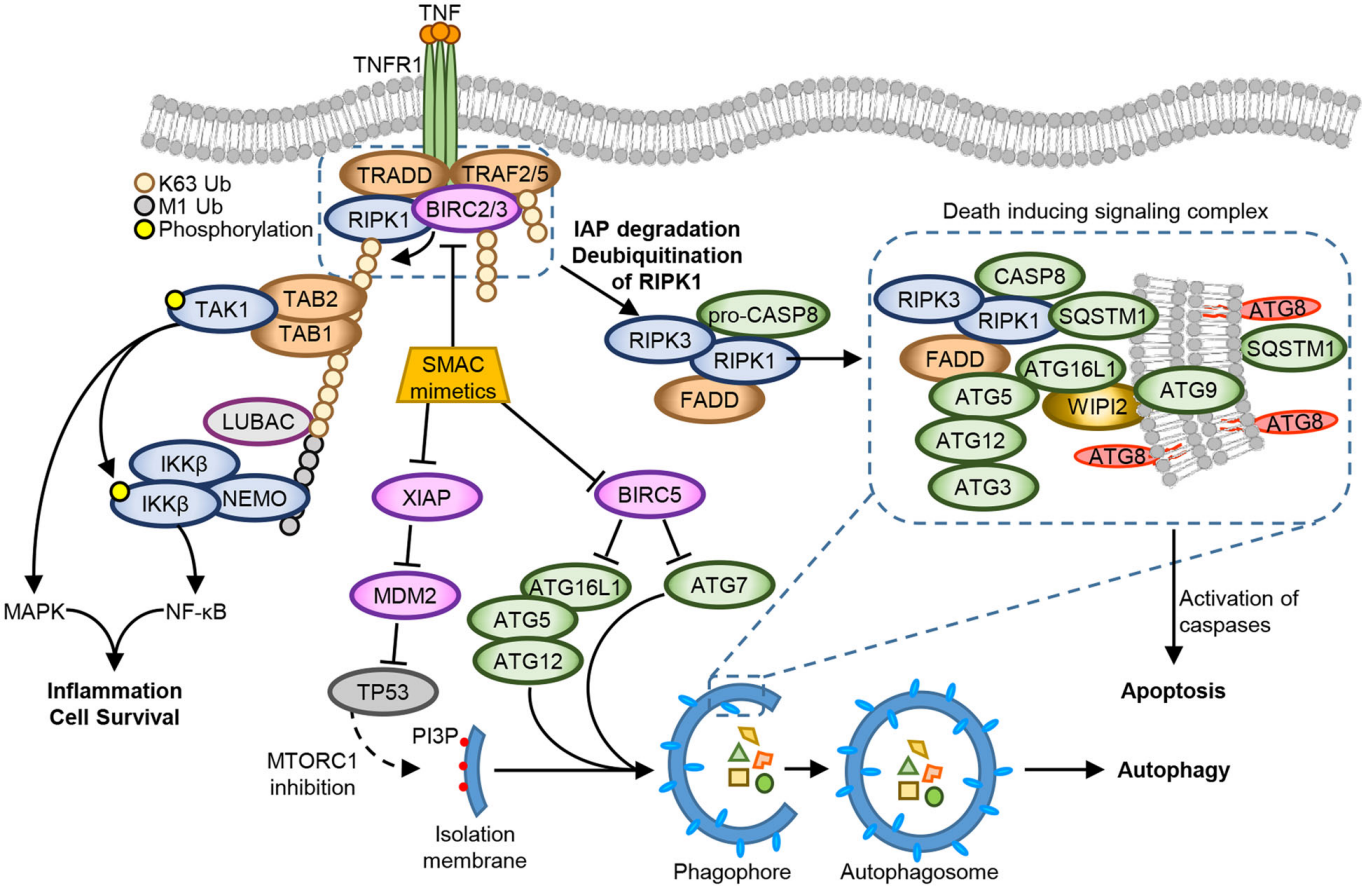
DIABO/SMAC mimetic mediated apoptosis. HIV‐1‐infected cells have increased expression of IAPs. Upon binding of TNF to TNFR1, RIPK1 is recruited to the receptor and is ubiquitinated by BIRC2 and BIRC3 and the LUBAC complex leading to the activation of MAPK signaling, inflammation, and cell survival. Separately, IAPs inhibit autophagy induction through the inhibition of MDM2 and the ATG12–ATG5‐ATG16L1 complex. Treatment of latent HIV‐infected cells with DIABLO/SMAC mimetics induces the degradation of IAPS. This results in the deubiquitination of RIPK1 and the induction of autophagy leading to the formation of a death inducing signaling complex on preautophagosome membranes, the activation of caspase‐8 and subsequent cell death by apoptosis

In addition to inducing autophagy, the DIABLO/SMAC mimetics induce the deubiquitination of RIPK1 in vitro in HIV‐1‐infected macrophages and latent HIV‐1‐infected resting memory CD4+ T cells but not uninfected cells. This deubiquitination leads to the formation of a DISC in HIV‐1‐infected cells consisting of FADD, RIPK1, RIPK3, activated caspase‐8, and the autophagy proteins ATG5, ATG7, and SQSTM1 (Figure 3).^68^, ^70^ The formation of this complex and the resultant selective killing of HIV‐1‐infected cells is dependent upon the initiation, nucleation, and formation of autophagosome phagophores, and the tethering of the DISC to these autophagosome phagophores via a RIPK1–SQSTM1 interaction. However, as the formation and activation of the DISC and selective cell death is not dependent upon autophagosome closure, autophago‐lysosome fusion, nor the degradation of the engulfed cargo(es), autophagy machinery is mediating DIABLO/SMAC mimetic‐induced apoptosis by serving as a scaffold and not through the degradation of cargo (Figure 4).^68^, ^70^, ^116^, ^117^ Moreover, the selective killing of resting HIV‐1‐infected memory CD4+ T cells was not dependent upon the release or addition of proinflammatory cytokines.^68^ Additionally, when encapsulated within uninfected CD4+ T cell plasma membrane wrapped poly(lactic‐*co*‐glycolic acid) (PLGA) core nanoparticles (TNP) the concentrations of the DIABLO/SMAC mimetics xevinapant and LCL‐161 required to induce autophagy‐dependent apoptosis of HIV‐1‐infected macrophages are lower, and the time required to induce apoptosis shorter.^115^ These TNP not only deliver cargo, but also have outstanding breadth and potency to neutralize cell‐free HIV‐1 (including broad‐spectrum global subtypes/recombinant forms as well as transmitted/founder viruses) and reduce cell‐associated HIV‐1 p24 through an autophagy‐dependent mechanism.^115^, ^118^, ^119^, ^120^ Importantly, although some DIABLO/SMAC mimetics can reactivate HIV‐1 from latent HIV‐1 cell lines through the activation of noncanonical NF‐κB signaling (Figure 4), only AZD‐5582, xevinapant, and ciapavir have demonstrated this ability in primary cells.^70^, ^117^, ^121^, ^122^, ^123^, ^124^ Notably, AZD‐5582 and ciapavir have undergone in vivo testing as LRAs in ART‐suppressed bone marrow–liver–thymus humanized mice infected with HIV‐1^123^, ^124^ and AZD‐5582 has undergone additional testing in rhesus macaques infected with SIV or SHIV.^122^, ^124^ These studies all indicated favorable pharmacokinetics and safety, highlighting the favorable safety profile for this class of drug. While ciapavir induced HIV‐1 transcription, AZD‐5582 showed mixed results as an LRA. Unfortunately, neither paper that demonstrated their efficacy as LRAs assessed the size of the inducible latent reservoir posttreatment.^123^, ^124^

Although not a DIABLO/SMAC mimetic, the BIRC5 inhibitor and autophagy inducer YM155 has also been shown to selectively kill in vivo HIV‐1‐infected reservoir CD4+ T cells encoding intact HIV‐1 and to decrease the frequency of clonally expanded HIV‐1 sequences.^67^ Additionally, the DDX3 inhibitors RK‐33 and FH‐1321 induce the down‐regulation of BIRC5 in HIV‐1‐infected cells ex vivo leading to the selective killing of HIV‐1‐infected primary CD4+ T cells.^97^ Collectively, these studies suggest that increased IAP expression during both productive and latent HIV‐1 infection sensitizes these cells to IAP inhibition, such that targeted pharmacologic inhibition of IAPs may lead to a reduction in the HIV‐1 reservoir.

### 3.7 Autosis‐inducing peptides

At doses that have no cytotoxic effects, the autophagy‐inducing peptide, Tat–beclin 1 (derived from amino acids 267–284 of BECN1 fused to amino acids 47–57 of HIV‐1 Tat), disrupts BECN1–GLIPR2 binding in the Golgi complex and inhibits the replication of HIV‐1.^125^ However, at increased doses, and when encapsulated within a PLGA nanoparticle, this peptide selectively kills in vitro latent HIV‐1‐infected primary CD4+ T cells through autosis.^119^ Similarly, the antiapoptotic α2‐helix derived from the death effector domain 1 of the K13 protein of human gammaherpesvirus 8 (v‐FLIP‐α2) that impairs FAS‐dependent CTL killing of HIV‐1‐infected cells and that can activate HIV‐1 transcription,^126^ also selectively induces autosis in in vitro HIV‐1‐infected primary resting memory CD4+ T cells and macrophages when encapsulated within a nanoparticle, with no deleterious effect on uninfected cells.^119^, ^127^ Nanoparticles are also being assessed as vehicles for antiviral drugs to improve drug tolerability, circulation half‐life, efficacy, and to target delivery to specific cells or anatomic compartments, to deliver cargo(es) that silence host and/or HIV‐1 gene expression, and to inhibit viral entry. ^115^, ^118^, ^120^, ^128^, ^129^ Thus, the emergence of nanomedicine has provided new avenues for HIV‐1 treatment and cure research, and the regulatory approval of lipid nanoparticle formulated drugs and vaccines highlights their promising future. However, to develop these nanoparticles as a treatment for HIV‐1, it is imperative to demonstrate their safety and efficacy in vivo.

## 4 CONCLUDING REMARKS

The development of a strategy that selectively kills latent HIV‐1‐infected cells while also reducing replication, transmission, and immune hyperactivation is the goal of HIV‐1 cure research. The “shock and kill” approaches have received the most attention, but to date their results are disappointing. However, the “prime, shock, and kill” strategy and the selective killing strategies described above still suffer from the same drawbacks as the “shock and kill” strategies. Namely, they do not address the heterogeneousness of the latent reservoir in various tissue compartments and sanctuary sites that have suboptimal drug penetration,^130^, ^131^ nor do they address the homeostatic proliferation of these reservoir cells, or the presence of noninducible HIV‐1 reservoirs.^61^ As HIV‐1 infection is highly complex and involves multiple cellular and tissue compartments, it is unlikely that a single agent approach will be successful. Despite this, the targeted killing of HIV‐1 reservoir cells is attractive, as it does not depend on the elicitation of a secondary immune response.

A number of important issues still need to be addressed through well‐designed preclinical and clinical trials. These include demonstrating efficacy and safety in vivo, defining the relationship between these multidrug regimens and residual viremia in tissues, including the CNS, assessing the ability of these drugs to penetrate the blood–brain barrier, assessing their effect on immune activation and/or chronic inflammation as well as off‐target effects, and whether escape mutations will evolve to avoid immunologic control. Despite these limitations, it is likely that targeted killing of HIV‐1‐infected cells will be involved in an HIV‐1 cure.

## AUTHORSHIP

G. R. C. wrote the manuscript. S. A. S. reviewed and edited the manuscript. All authors read and approved the final version of the manuscript.

## DISCLOSURE

The authors declare that they have no commercial or financial relationships that could be construed as a potential conflict of interest.
